# Common Endpoints in Studies of Image‐Based Guidance in External Ventricular Drain Placement: A Scoping Review

**DOI:** 10.1002/brb3.71432

**Published:** 2026-04-22

**Authors:** Andrew Bohner, Lucas Garfinkel, Kaitlyn Piotrowski, Aizhan Mengaliyeva, Manisha Koneru

**Affiliations:** ^1^ Cooper Medical School of Rowan University Camden New Jersey USA; ^2^ Cooper Neurological Institute Cooper University Health Care Camden New Jersey USA

**Keywords:** cerebral ventricles, clinical trials, device safety, drain, neurological diagnostic techniques

## Abstract

**Purpose:**

External ventricular drain placement (EVD), is a widely used procedure utilized to relieve intracranial pressure due to a variety of pathological conditions, such as hemorrhage or hydrocephalus. EVD placement is generally performed at the bedside using a freehand technique. This can lead to malpositioning of the catheter tip, especially in patients with pathologies that result in displacement of the lateral ventricles, midline shift, and a low bicaudate index. Novel developments in technology have allowed for image‐guided navigation systems to be implemented into EVD placement procedures, reducing the risks for malpositioning and potential complications. This scoping review aims to identify common outcomes and design elements within prospective studies focused on comparing image‐guided EVD placement to the freehand technique.

**Method:**

ClinicalTrials.gov was queried for EVD placement trials evaluating image guidance techniques in at least one arm. Prevalence of study design were summarized. Results were reported in accordance to PRISMA guidelines.

**Finding:**

Of the seven studies included, most studies had one week of follow‐up data to ascertain endpoints, and compared image‐guided versus freehand approaches. The most common outcomes of importance were Kakarla grade for EVD tip placement and the number of passes required. Studies commonly emphasized characterizing accuracy of placement and procedural efficiency.

**Conclusion:**

Although challenges remain to optimize these methods and standardize research protocols, future studies incorporating standardized endpoints and addressing current gaps in existing studies will help drive conduct of studies well‐designed to improve patient outcomes.

## Graphical abstract

When exploring EVD placement safety, accuracy, and efficiency comparing image‐guided techniques and freehand techniques, incorporating standardized endpoints and addressing gaps from past studies will help guide the design of future studies to optimize results translatable to improving patient outcomes.

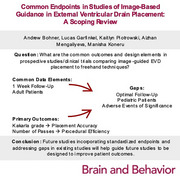

## 1 Introduction

External ventricular drain (EVD) placement is a common neurosurgical procedure indicated for cerebrospinal fluid diversion to alleviate high intracranial pressures due to pathologies, such as traumatic brain injury, hydrocephalus, and hemorrhage (Palasz et al. 2022; Addis et al. 2023; Taylor et al. 2024). Using external anatomical landmarks and a freehand technique is the current standard of care; however, there is a risk of multiple passes or malpositioning, increasing the likelihood of post‐placement hemorrhages (Maher Hulou et al. 2022; Khalaf et al. 2020; Charcos et al. 2025). Successful EVD placement can vary widely when using the freehand technique due to pathologic anatomical changes, making accurate placement challenging (Maher Hulou et al. 2022).

Recently, image‐guided navigation systems have been introduced as an adjunct, utilizing pre‐EVD imaging to guide catheter placement to prevent malpositioning and improve accuracy (Charcos et al. 2025; Aljoghaiman et al. 2022; Fisher et al. 2021). Several different image‐guided solutions have been proposed as alternatives to the freehand method, with a focus on speed and usability (Aljoghaiman et al. 2022; Grunert et al. 2024; van Gestel et al. 2021; Ofoma et al. 2022). Prospective studies have evaluated the experience of providers utilizing the image‐guidance technology, as well as comparing outcomes amongst freehand and image‐guidance groups. Similar to other studies evaluating the safety and efficacy of new adjunctive tools and methods in neurovascular care, there is wide variation in approaches to conducting these prospective studies (Koneru et al. 2025). In this review, we aim to summarize the design elements and outcomes common to the prospective studies conducted thus far comparing image‐guided EVD placement and the current standard of care. Our objective in summarizing these design elements and outcomes is to characterize the current trends to inform norms for future studies, identify gaps in the studies performed, and ultimately standardize approaches for image‐guidance EVD placement evaluation.

## 2 Methods

Data may be available upon reasonable request. This review is exempt from institutional ethical review board approval; data for this scoping review was reported in accordance with the Preferred Reporting Items for Systematic Reviews and Meta‐Analyses guidelines (Tricco et al. 2018).

A query of Clinicaltrials.gov was conducted for studies added to the database before or on December 25, 2024 with the following query: “(EVD OR external ventricular drain OR external ventricular drainage OR ventriculostomy) AND (navigation OR image OR guidance).” Studies retrieved from the query were screened by two independent reviewers based on criteria for inclusion and exclusion, with a third reviewer's input to resolve discordant decisions (External Ventricular Drain Placement Stealth Study [NCT03696043] 2025; Image Guided EVD Placement [NCT05639036] 2025; Augmented Reality‐Assisted Neurosurgical Drain Placement [ARANED] [NCT06571539] 2025; VisAR Augmented Reality Navigation of Ventriculostomy [NCT06132139] 2025; Ultrasound [US] Guided External Ventricular Catheter Placement [NCT06253858] 2025; Tablet‐guided Versus Freehand [Tab‐Guide] Ventriculostomy: Study Protocol to the Test Accuracy of Ventriculostomy in a Randomized Controlled Trial [Tab‐Guide] (NCT02048553] 2025; Intraventricular Drain Insertion: Comparison of Ultrasound‐guided and Landmark‐Based Puncture of the Ventricular System [In‐Vent] [NCT01973764] 2025). For inclusion, studies must have: (1) been focused on evaluating EVD placement into the cranium at the bedside, and (2) used an image guidance for placement in at least one study arm. Studies at any stage (i.e., active, completed, unknown) were included. Full‐text review of the Clinicaltrials.gov entry page performed by a single reviewer extracted the following common data elements: Number of patients, maximum follow‐up duration, type of study population, type of comparator arm, and various outcomes or endpoints of interest (e.g., accuracy of catheter tip placement, success of placement, number of passes, revision, subjective user experience, or complications).

### 2.1 Statistical Analysis

The primary outcome was the proportion of study design and endpoints shared across included image‐guided EVD studies. Extracted data was summarized with medians with interquartile ranges (IQR) or frequencies using JMP version 18.0 (SAS Institute, Cary, NC).

## 3 Results

**FIGURE 1:**
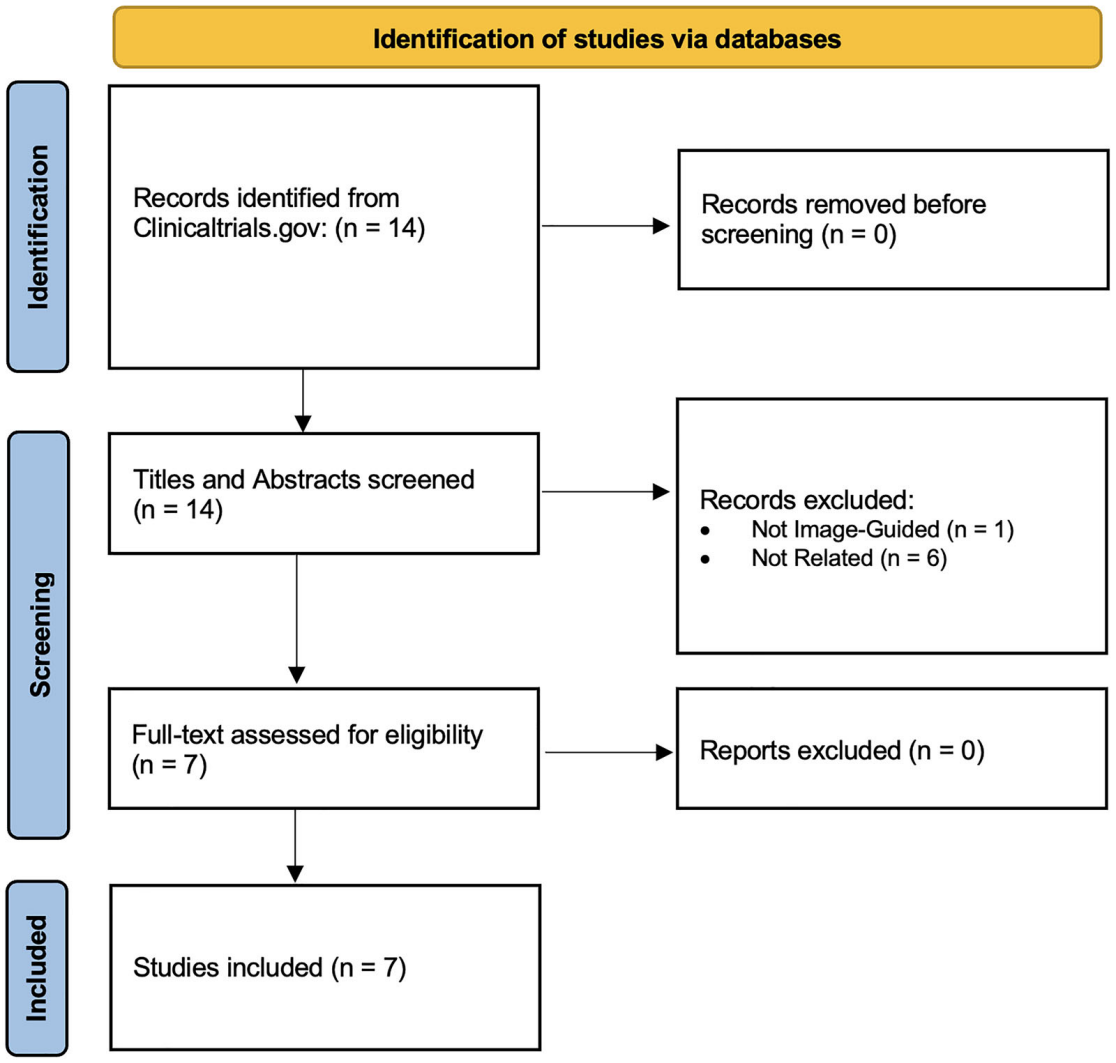
Flowchart of included studies.

Of the seven studies included, the median number of patients included was 50 (IQR 22–100); all studies were conducted in adults (Tables 1 and 2; Figure 1). Most studies required pre‐EVD placement imaging (i.e., either computed tomography or magnetic resonance imaging of the brain) (5/7, 71.4%), and two studies used ultrasound‐guidance (2/7, 28.6%) (Table 1). Most studies were comparing image‐guided EVD placement to freehand EVD placement rather than to another image‐guidance platform (6/7, 85.7%) (Table 2).

**TABLE 1:** Included studies.

National Clinical Trial identifier number	Study title	Image‐guided system
NCT03696043	External Ventricular Drain Placement Stealth Study	AxiEM Stealth Image Guidance (Medtronic, Minneapolis, MN)
NCT05639036	Image Guided EVD Placement	Stryker Nav3 Image Guidance System (Stryker, Kalamazoo, MI)
NCT06571539	Augmented Reality‐Assisted Neurosurgical Drain Placement	Augmented Reality Guidance for EVD
NCT06132139	VisAR Augmented Reality Navigation of Ventriculostomy	VisAR Augmented Reality Surgical Navigation (Novarad, Provo, UT)
NCT06253858	Ultrasound (US) Guided External Ventricular Catheter Placement	Solopass US system (InTRAvent, Hershey, PA)
NCT02048553	Tablet‐Guided Versus Freehand (Tab‐Guide) Ventriculostomy: Study Protocol to the Test Accuracy of Ventriculostomy in a Randomized Controlled Trial	Custom Mini‐Tablet Application
NCT01973764	Intraventricular Drain Insertion: Comparison of Ultrasound‐Guided and Landmark‐Based Puncture of the Ventricular System	Ultrasound

Abbreviation: EVD = External ventricular drain, US = Ultrasound.

**TABLE 2:** Study design features.

Study features	*n* = 7 studies
Number of total patients, median (IQR)	50 (22–100)
Number of patients in image‐guided EVD arm, median (IQR)	25 (11–50)
Maximum length of follow‐up (days), median (IQR)	15.5 (7.8–27.8), *n* = 4
Age of study population, no. (%)	
Adults only	7 (100%)
Pediatrics only	0 (0%)
Type of comparator arm, no. (%)	
Freehand EVD placement	6 (85.7%)
No comparator	1 (14.2%)

Abbreviation: EVD = External ventricular drain, IQR = Interquartile.

The most common endpoint for assessment was the number of attempted passes before achieving adequate EVD placement (6/7, 85.7%) (Table 3). Most studies assessed placement efficacy by characterizing the accuracy of catheter tip placement reaching the target site (5/7, 71.4%) and the number requiring revision (5/7, 71.4%) (Table 3). Accuracy of tip placement was most commonly assessed using the Kakarla Grade (5/7, 71.4%) (Table 3). Complications intra‐procedurally and in the follow‐up period were also commonly reported (6/7, 85.7%), although the type of complication varied widely between studies, including any non‐specific complication, having the EVD be pulled out, post‐placement hemorrhage (a generally acute EVD complication), and post‐placement infection (a generally delayed EVD complication) (Table 3).

**TABLE 3:** Reported endpoints of interest.

Endpoint	*n* = 7 studies
Accuracy of catheter tip, no. (%)	5 (71.4%)
Accuracy of tip with Kakarla grade, no. (%)	5 (71.4%)
Accuracy of tip via anatomical landmark, no. (%)	3 (42.9%)
Number of attempts/passes before placement, no. (%)	6 (85.7%)
Placement success, no. (%)	3 (42.9%)
Time required for placement, no. (%)	2 (28.6%)
Requiring EVD revision, no. (%)	5 (71.4%)
New neurological symptoms, no. (%)	1 (14.3%)
User experience, no. (%)	1 (14.2%)
Type of complication(s) as endpoints, no. (%)	
Any	6 (85.7%)
EVD Was pulled out	0 (0%)
Post‐placement hemorrhage	2 (28.6%)
Post‐placement infection	3 (42.9%)
Non‐specific	2 (28.6%)

Abbreviation: EVD = External ventricular drain.

## 4 Discussion

We summarized the common data elements for study design and outcome assessment for evaluating image‐guided EVD placement techniques. Most studies evaluating EVD placement typically compare image‐guided techniques with the traditional free‐hand method to assess for differences in accuracy and efficiency. In addition, a follow‐up period of at least 1–2 weeks is often included to best monitor for both acute (i.e., post‐placement hemorrhage) and delayed complications (i.e., post‐placement infection). The key efficacy outcomes measured commonly include the Kakarla grading system for assessing tip placement and the number of passes required for successful insertion. These metrics facilitate assessment of overall optimization of the EVD placement process by characterizing procedural efficacy and efficiency (Fisher et al. 2021). Optimal placement per Kakarla grading has been associated with better EVD placement and increased likelihood of clinical improvement from EVD; reduction in the number of passes for EVD placement has been associated with fewer iatrogenic complications (Fisher et al. 2021). Consequently, in addition to procedural efficacy, the key outcomes commonly shared amongst most prospective studies are also markers of improved patient outcomes.

In order to enhance clinical relevance and comparability, it is recommended that studies incorporate these common design elements as standardized endpoints in future research on EVD placement techniques. This approach will help ensure that findings are more consistent and directly applicable across different studies, ultimately improving scope to make adequate assessments regarding the translational impact of image‐guidance in studies aimed at optimizing EVD placement outcomes.

Despite advancements in image‐based guidance for EVD placement, there are several areas of improvement to optimize patient outcomes and advance the field. Future studies may consider sub‐stratifying the patient cohort by anatomical features on baseline imaging that would be indicative of a more challenging EVD placement, such as midline shift and mass effect causing ventricle displacement, in order to further specifically assess performance of these approaches in both straightforward and difficult situations. Future studies may also incorporate evaluation of alternative adjunct tools, such as a Fechar gauge for triangulation, that are not as laborious to set‐up as image‐guidance systems but do provide more visual guidance than the freehand technique. Incorporating similar practical tools within the scope of future studies and real‐world practice will facilitate identification of adjunct tools that balance simplicity, efficiency, and accuracy.

Moreover, current studies often lack consistent pre‐definition and assessment of complications such as hemorrhage, infection, and malfunction, highlighting the need for standardized criteria for complications of interest. For example, ischemic stroke endovascular thrombectomy studies often assessed and reported post‐thrombectomy hemorrhage according to the European Cooperative Acute Stroke Study III (ECASS‐II) classification to standardize assessment regarding degree and severity of hemorrhage (Lakhani et al. 2025; Yedavalli et al. 2024; Koneru et al. 2024). Similarly, standardized approaches to assessing complications are particularly important in the context of prospective studies for EVD placement, as this practice will help aid efficient safety monitoring and facilitate early intervention to prevent additional complications (Gibas et al. 2024; Hanley et al. 2023; Haldrup et al. 2023). Variable follow‐up intervals further challenge research, underscoring the importance of determining optimal durations to effectively capture necessary complications without unnecessarily prolonging study timelines. In addition, there is a significant gap in research involving pediatric populations, a group with unique anatomical variations that demand tailored technologies and rigorous evaluation (LoPresti et al. 2018). User experience is another underexplored area, as the adoption of new image‐guided technologies often involves a steep learning curve for physicians (Glas et al. 2021; Lin et al. 2023; Ljungqvist et al. 2024). Incorporating endpoints that evaluate physician experience and satisfaction both within a clinical trial setting and through pragmatic observational studies could provide valuable insights into barriers to implementation and inform the development of training programs. Addressing these challenges will enhance both the quality of research and the standard of care.

## 5 Conclusions

Studies examining image‐guided techniques for EVD placement commonly emphasize characterizing accuracy of placement and procedural efficiency. However, challenges remain in optimizing these methods and standardizing research protocols. Future research should standardize endpoints, define complications clearly, ensure consistent follow‐up, and assess user experience. More studies are also needed in pediatric populations and to evaluate the learning curve of new technologies. Future studies incorporating standardized endpoints and addressing current gaps in existing studies will help drive conduct of studies well‐designed to improve patient outcomes, optimize clinical practices, and support training programs, ultimately advancing neurosurgical care and long‐term patient results.

## Author Contributions

**Andrew Bohner**: conceptualization; investigation; writing – original draft; writing – review and editing; data curation. **Lucas Garfinkel**: investigation; data curation; writing – original draft; writing – review and editing. **Kaitlyn Piotrowski**: data curation; writing – original draft; writing – review and editing; investigation. **Aizhan Mengaliyeva**: data curation; writing – original draft; writing – review and editing; investigation. **Manisha Koneru**: writing – review and editing; writing – original draft; data curation; conceptualization; methodology; investigation; supervision; project administration; visualization.

## Funding

The authors have nothing to report.

## Ethics Statement

This scoping review was exempt from institutional review board review.

## Consent

The authors have nothing to report.

## Conflicts of Interest

The authors declare no conflicts of interest.

## Data Availability

Data may be made available upon reasonable request to the corresponding author.
